# HIV-1 Residual Viremia Correlates with Persistent T-Cell Activation in Poor Immunological Responders to Combination Antiretroviral Therapy

**DOI:** 10.1371/journal.pone.0007658

**Published:** 2009-10-30

**Authors:** Maud Mavigner, Pierre Delobel, Michelle Cazabat, Martine Dubois, Fatima-Ezzahra L'Faqihi-Olive, Stéphanie Raymond, Christophe Pasquier, Bruno Marchou, Patrice Massip, Jacques Izopet

**Affiliations:** 1 INSERM, U563, Toulouse, France; 2 Université Toulouse III Paul-Sabatier, Centre de Physiopathologie de Toulouse Purpan, Toulouse, France; 3 CHU Toulouse, Hôpital Purpan, Service des Maladies Infectieuses et Tropicales, Toulouse, France; 4 CHU Toulouse, Hôpital Purpan, Laboratoire de Virologie, Toulouse, France; University of California San Francisco, United States of America

## Abstract

**Background:**

The clinical significance and cellular sources of residual human immunodeficiency virus type 1 (HIV-1) production despite suppressive combination antiretroviral therapy (cART) remain unclear and the effect of low-level viremia on T-cell homeostasis is still debated.

**Methodology/Principal Findings:**

We characterized the recently produced residual viruses in the plasma and short-lived blood monocytes of 23 patients with various immunological responses to sustained suppressive cART. We quantified the residual HIV-1 in the plasma below 50 copies/ml, and in the CD14^high^ CD16^−^ and CD16^+^ monocyte subsets sorted by flow cytometry, and predicted coreceptor usage by genotyping V3 *env* sequences.

We detected residual viremia in the plasma of 8 of 10 patients with poor CD4^+^ T-cell reconstitution in response to cART and in only 5 of 13 patients with good CD4^+^ T-cell reconstitution. CXCR4-using viruses were frequent among the recently produced viruses in the plasma and in the main CD14^high^ CD16^−^ monocyte subset. Finally, the residual viremia was correlated with persistent CD4^+^ and CD8^+^ T-cell activation in patients with poor immune reconstitution.

**Conclusions:**

Low-level viremia could result from the release of archived viruses from cellular reservoirs and/or from ongoing virus replication in some patients. The compartmentalization of the viruses between the plasma and the blood monocytes suggests at least two origins of residual virus production during effective cART. CXCR4-using viruses might be produced preferentially in patients on cART. Our results also suggest that low-level HIV-1 production in some patients may contribute to persistent immune dysfunction despite cART.

## Introduction

Treatment of human immunodeficiency virus type-1 (HIV-1) with combined antiretroviral therapy (cART) decreases the plasma HIV-1 RNA load to below the detection limit of standard assays (<50 copies/ml), resulting in dramatic improvements in the clinical course of HIV-1 infection. However HIV-1 cannot be eradicated from infected individuals by current regimens and low levels of HIV-1 RNA in the plasma can be detected using extremely sensitive reverse transcriptase assays, even in patients on prolonged effective cART [1]–[7]. This residual viremia may indicate active virus production, as the half life of free virus particles is short [8], [9]. The cellular sources and the clinical significance of persistent low-level viremia below 50 copies per ml of plasma are not well-defined. The residual viremia could be due to low-level replication that continues despite cART [3], [10]–[14] and/or the release of viruses from latently infected cells [14]–[18].

While resting memory CD4^+^ T-cells are the most significant cellular reservoir for HIV-1 [19]–[25], other reservoirs can contribute to virus persistence. Poor penetration or the active efflux of antiretroviral drugs may allow the virus to continue replicating in certain anatomical or cellular compartments. During cART, HIV-1 has been found to persist in cells such as naïve CD4^+^ T-cells [19], [26]–[28], tissue macrophages [29], [30], and peripheral blood monocytes [31]–[35]. Blood monocytes, derived from mononuclear phagocytic precursor cells in the bone marrow, may circulate in the peripheral blood for one to three days before they differentiate into immature dendritic cells and tissue macrophages [36], [37]. Thus, persistent HIV-1 in blood monocytes suggests recent infection and/or ongoing replication in monocytes or in their precursor cells. Indirect evidence for ongoing virus replication in patients on suppressive cART also includes the detection of unintegrated HIV-1 DNA [21], [38], [39], cell-associated HIV-1 RNA [11], [12], [32], [40], and significant genetic evolution of HIV-1 sequences [40]–[42].

The residual replication of HIV-1 in patients on cART could allow genetic evolution of the virus quasispecies, and changes in virus coreceptor use. Our previous five-year longitudinal study found that CXCR4-using HIV-1 could be gradually selected in cellular reservoirs during sustained effective cART, and that the presence of CXCR4-using viruses could be associated with impaired CD4^+^ T-cell restoration [43]. Because of the differential expression of HIV-1 coreceptors CCR5 and CXCR4 on distinct T-cells subsets [44], the residual replication of CCR5- and CXCR4-using viruses could have different impacts on T-cell homeostasis during immune reconstitution on cART. The interplay between virus tropism and CD4^+^ T-cell restoration on cART was previously examined by comparing the virological and immunological features of two groups of patients, one having poor CD4^+^ T-cells restoration despite a sustained virological response and the second having good immunological and virological responses (ANRS EP32 study) [28]. This study suggests that the persistent CD4^+^ T-cell lymphopenia in the poor immunological responders was mainly due to persistent T-cell activation and apoptosis on cART, and this was associated with a high frequency of CXCR4-using viruses in the peripheral blood mononuclear cells (PBMCs) of the poor immunological responders.

We have now analyzed the residual production of HIV-1 in patients on effective cART by characterizing the plasma and monocyte compartments of 23 patients from the ANRS EP32 study. We measured residual plasma viremia below 50 copies/ml using an ultrasensitive assay with a detection limit of 2.5 copies/ml and characterized coreceptor use by amplifying V3 *env* with the same detection sensitivity. The residual HIV-1 sequences found in the plasma were compared to the sequences in CD14^high^ CD16^−^ and CD16^+^ monocytes and CD4^+^ T-cells. Finally, we assessed whether residual HIV-1 viremia could have a significant impact on the T-cell homeostasis of patients on sustained cART, and particularly whether it could be related to the persistent T-cell activation that occurs in the poor immunological responders.

## Materials and Methods

### Study subjects and samples

Subjects were selected from HIV-1 infected patients characterized in the ANRS EP32 study [28]. This study compared two groups of 15 patients, one having poor CD4^+^ T-cell restoration (gain of <200 CD4^+^ T-cells/µl) despite a sustained virological response and the second having good immunological (gain of >500 CD4^+^ T-cells/µl) and virological responses. We excluded two patients in each group because of non-subtype B HIV-1 infection, and three additional patients in the poor immunological responder group because no cryopreserved plasma samples were available. We thus studied 10 poor immunological responders and 13 good immunological responders. All of these patients had been on cART for a median of 84 months at the time of analysis (interquartile range, IQR [60–94]), and had had a sustained undetectable plasma virus load throughout follow-up. The patient characteristics are shown in Table S1 in the supplemental material. We analysed cryopreserved samples taken before these patients began cART and while they were on effective cART to characterize the residual HIV-1 in the plasma and the monocyte and CD4^+^ T-cell compartments.

### Ethics Statement

This research was approved by the Institutional Review Boards of Toulouse University Hospital and the Pasteur Institute of Paris, France. Written informed consent was obtained from all the participants in this study.

### Characterization of HIV-1 residual viremia below 50 copies/ml of plasma

#### Viral RNA extraction

Plasma samples (12 ml) were centrifuged to remove contaminating cells and then ultracentrifuged at 20 000 g for 75 min at 4°C to collect the virus particles. Viral RNA was then isolated using the QIAamp UltraSens Virus Kit (Qiagen). Half of the extracted RNA (equivalent to 6 ml of plasma) was used to quantify HIV-1 by real-time RT-PCR; the other half was used to amplify V3 *env* by nested RT-PCR and so predict coreceptor use.

#### Quantification of residual plasma HIV-1

Residual plasma virus was quantified from the RNA extracted from 6 ml of plasma using an ultrasensitive real-time RT-PCR assay with a detection limit of 2.5 copies/ml. The LightCycler RNA master Hybprobe kit (Roche diagnostics) was used to amplify conserved sequences within the LTR region using the primers 5′-GGCGCCACTGCTAGAGATTTT-3′ and 5′-GCCTCAATAAAGCTTGCCTTGA-3′ and a Taqman probe 5′-6Fam-AAGTAGTGTGTGCCCGTCTGTTRTKTGACT-Tamra-3′. The amplification mix contained 1× LightCycler RNA master Hybprobe, 3.25 mM of Mn(OAc)_2_, 0.3 Unit of Uracil DNA Glycosylase, 0.5 µM of each primer and 0.25 µM of the Taqman probe. RT-PCR was performed with the following conditions: 30 min at 59°C; 30 s at 95°C, 5 s at 95°C and 45 s at 62°C for 50 cycles on a LightCycler (Roche diagnostics).

Synthetic HIV-1 RNA was used to generate a standard curve. Briefly, a 190 bp fragment of the LTR region of HIV-1 HXB2 was inserted into the pGEM-3Z vector downstream of the T7 promoter. The pGEM-3Z recombinant plasmid was linearized with SmaI, and transcribed with T7 RNA polymerase using the Riboprobe In Vitro Transcription System (Promega). The template DNA was digested with RNase-free DNase, and the RNA transcripts were purified with an RNeasy kit (Qiagen). Serial dilutions of synthetic HIV-1 RNA were used to generate a standard curve for each assay. Any DNA contamination was excluded by performing PCR amplifications without the reverse transcription step in four patients with detectable residual plasma virus. The sensitivity of the assay was assessed using plasma samples containing known copy numbers of HIV-1 (NIBSC HIV-1 RNA second international standard diluted in HIV-1-seronegative human plasma).

#### Determination of the coreceptor usage of residual plasma HIV-1

The coreceptor usage of the residual virus was determined genotypically in parallel with its quantification. A region spanning the V3 region of HIV-1 *env* was amplified from the RNA extracted from 6 ml plasma using an ultrasensitive conventional nested RT-PCR assay with a detection limit of 2.5 copies/ml. RT-PCR was performed with the One-Step RT-PCR kit (Qiagen), with the following conditions: 30 min at 50°C; 15 min at 95°C; 10 s at 94°C, 30 s at 55°C, and 1 min at 72°C for 50 cycles. Nested PCR was performed with the Expand High Fidelity Plus PCR System (Roche), with the following conditions: 2 min at 94°C; 30 s at 94°C, 30 s at 55°C, and 30 s at 72°C for 50 cycles. PCRs were performed using 5′-ACAATGYACACATGGAATTARGCCA-3′ and 5′-TTTAATTGTYGAGGYGAATTTTTCT-3′ as outer primers and 5′- CTGTTAAATGGCAGTCTAGC-3′ and 5′ CCCCTCCACAATTAAAACTGT-3′ as inner primers on a GeneAmp PCR System 9700 (Applied Biosystems). At least 10 individual molecular clones obtained using the TOPO-TA cloning kit (Invitrogen, Cergy Pontoise, France) were sequenced in both directions by the dideoxy chaintermination method (Big Dye Terminators v.3.1, Applied Biosystems, Paris, France) on an 3130xl Genetic Analyzer (Applied Biosystems). Contamination with DNA was ruled out by performing PCR amplifications without the reverse transcription step in four patients with detectable residual plasma virus.

We predicted HIV-1 coreceptor usage from the V3 genotype using a combination of criteria from the 11/25 and net charge rules, as previously described [45], [46].

### Characterization of residual HIV-1 in CD14^high^ CD16^−^ and CD16^+^ monocytes

#### Isolation of CD14^high^CD16^−^ and CD16^+^ monocyte subsets

Cryopreserved PBMCs (15-25.10^6^ cells) were stained with PECy7-conjugated anti-CD3, ECD-conjugated anti-CD4, PE-conjugated anti-CD56, AlexaFluor488-conjugated anti-CD14, and PECy5-conjugated anti-CD16 mAbs (all mAbs were from BD Pharmingen except the CD3- PECy7 and CD4-ECD mAbs, which were from Beckman Coulter). Cells were sorted by flow cytometry on an Epics ALTRA (Beckman-Coulter). Purities were determined by flow cytometry analysis of the sorted cells on the same instrument with the same instrument settings. The sorted cells populations were routinely >99% pure.

#### Quantification of HIV-1 DNA in the monocyte subsets

A real-time PCR assay was used to quantify the HIV-1 DNA load (*gag* region) from monocyte lysates using fluorescence resonance energy transfer probes on a LightCycler (Roche Diagnostics), as previously described [28]. This quantitative PCR assay detects one copy of HIV-1 DNA in 10^5^ cells.

#### Determination of the coreceptor usage of HIV-1 in the monocyte subsets

A nested PCR was used to amplify a region spanning the V3 region of HIV-1 *env* from monocyte-associated DNA. Both primary and nested PCRs were performed using the Expand High Fidelity Plus PCR system (Roche Diagnostics, Meylan, France). The PCR products from three parallel amplifications were pooled to prevent sampling bias and cloned using the TOPO-TA cloning kit (Invitrogen, Cergy Pontoise, France) and at least 10 individual molecular clones were sequenced. HIV-1 coreceptor usage was predicted from the V3 genotype using a combination of criteria from the 11/25 and net charge rules, as previously described [45], [46].

### Phylogenetic analysis

Multiple alignments were performed with the CLUSTAL W program, and sequence alignments were edited manually using BioEdit software. Dendograms were created by the neighbor-joining method with the Phylogeny Interference Package (PHYLIP) and tree diagrams were plotted with the TREEVIEW 1.6.6 program. The HXB2 sequence (GenBank K03455) was included in all phylogenetic analyses as an outgroup. Maximum likelihood phylogenies with the same sequence datasets were also estimated using PhyML 3.0, providing similar tree topologies (data not shown). The most appropriate nucleotide substitution model for maximum likelihood phylogenetic analysis was determined with the model selection procedure implemented in the program Modeltest 3.7. Bootstrap analysis consisting of 100 replicates was performed on the phylogenetic trees. Phylogenetic analysis of V3 sequences from the entire PCR products showed distinct clusters of sequences for each patient that excluded any possibility of sample contamination or mix-up (data not shown).

### Immunological analysis

#### Sorting of naive T-cell subsets

The CD31^+^ and CD31^−^ subsets of CD45RA^+^ CD27^+^ CD4^+^ T-cells, and the CD45RA^+^ CD27^+^ CD8^+^ T-cell subset had been sorted in the ANRS EP 32 study by flow cytometry from cryopreserved PBMCs on a MoFlo cell sorter (Dako-Cytomation, Trappes, France). The sorted cell populations were routinely better than 99% pure [28].

#### Flow cytometry analysis

We used the immunophenotypic analyses that had been previously performed in the ANRS EP 32 study by flow cytometry from cryopreserved PBMCs to measure the proportion of activated cells among CD4^+^ and CD8^+^ T-cells using the HLA-DR and CD38 markers, and the proportion of CD31^high^ cells among the CD45RA^+^ CD27^+^ naïve CD4^+^ T-cells [28]. Flow cytometric acquisition (0.5–5×10^5^ events) and analysis were performed on a Cytomics FC500 driven by the RxP sofware package (Beckman-Coulter).

#### TRECs quantification

The sjTREC (δRec-ψJα) and each of the 10 DβJβTRECs (Dβ1-Jβ1.1 to Dβ1-Jβ1.6 and Dβ2-Jβ2.1 to Dβ2-Jβ2.4) had been measured in the ANRS EP 32 study by nested quantitative PCR [28]. The sjTREC was quantified in triplicate and each of the 10 DβJβTRECs in duplicate. The sum of the DβJβTREC frequencies is 1.3-times the sum of the 10 measured DβJβTREC frequencies (in order to extrapolate to the 13 principal DβJβTRECs). The sj/βTREC ratio is the sjTREC frequency divided by the sum of DβJβTREC frequencies [47].

### Statistical analysis

Quantitative and categorical variables were compared using the Wilcoxon rank sum test and Fisher's exact test, respectively. Correlations between quantitative variables were estimated by calculating Spearman's rank correlation coefficient. All tests were two-tailed, and *P* values of <0.05 were considered statistically significant. Statistical analyses were performed with Stata SE 9.2.

### Nucleotide sequence accession numbers

The sequences reported here were given Genbank accession numbers GU063135 to GU063691.

## Results

### HIV-1 DNA in CD16^+^ and CD14^high^ CD16^−^ monocyte subsets

Blood monocytes consist of a major CD14^high^ CD16^−^ subset and a minor CD16^+^ subset with distinct phenotypic and functional characteristics [48]–[50]. CD16^+^ monocytes express more CCR5 on their surface than do CD14^high^ CD16^−^ monocytes [51], [52], and it has been suggested that the CD16^+^ subset could be preferentially infected by HIV-1 [51], [53]. We sorted the CD14^high^ CD16^−^ and CD16^+^ monocyte subsets of the 23 patients from their PBMCs by flow cytometry (see Fig. S1 for cell sorting of blood monocyte subsets in the supplemental material). The median percentage of CD16^+^ monocytes was 15.3% (IQR, [11.6–18.4]) of the total monocytes in the poor immunological responders, and 13.3% (IQR, [11.9–17.8]) in the good immunological responders (*P* = 0. 90).

The short half-life of monocytes in the peripheral blood suggests that any HIV-1 detected in these cells was due to recent infection and/or ongoing replication in monocytes or in their precursor cells despite cART. We used nested PCR to amplify a region spanning the V3 region of HIV-1 *env* in the sorted monocyte subsets. Three amplifications were performed in parallel and pooled to prevent sampling bias. These data were used for genotypic prediction of coreceptor usage and phylogenetic analyses. We report the detection of HIV-1 in the sorted monocyte subsets only for patients for whom PCR was done on >10^5^ sorted cells, *i.e.* all of the 23 patients for the majority CD14^high^ CD16^−^ monocyte subset, but only seven patients for the minority CD16^+^ monocyte subset. HIV-1 DNA was detected in the CD14^high^ CD16^−^ monocytes from five of the 23 patients (patients 15, 16, 18, 19 and 29), and in the CD16^+^ monocytes from two of the seven analyzable patients (patients 22 and 29). One patient (patient 29) had detectable HIV-1 in both monocyte subsets. We also used real-time PCR with primers that amplify a highly conserved region of *gag* to quantify the HIV-1 DNA in the monocyte subsets. The detectable HIV-1 DNA was always <10 copies per 10^6^ cells, without any significant differences between the monocyte subsets (data not shown).

### Compartmentalization of HIV-1 between CD14^high^ CD16^−^ monocytes, CD16^+^ monocytes, and CD4^+^ T-cells from patients on effective cART

**Figure 1:**
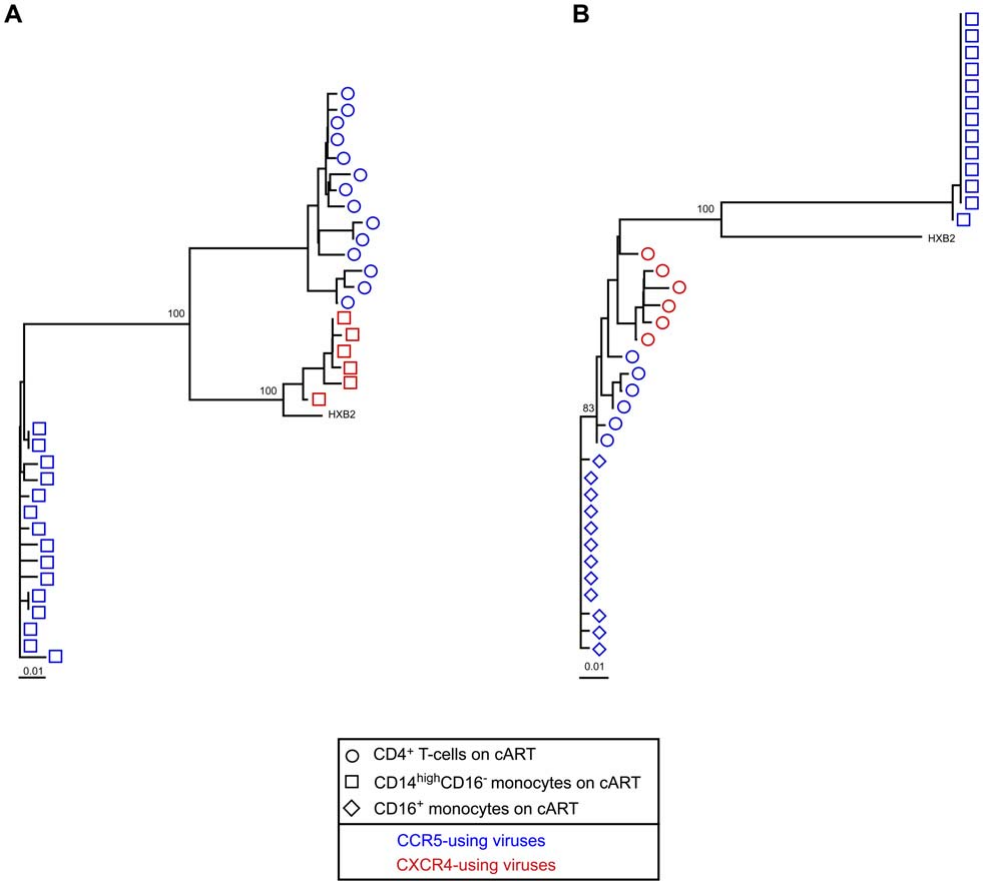
HIV-1 compartmentalization between CD14^high^ CD16^−^ and CD16^+^ monocytes. Phylogenetic trees of HIV-1 *env* sequences obtained by clonal analysis of PCR products from CD14^high^ CD16^−^ monocytes (open squares), CD16^+^ monocytes (open diamonds), and CD4^+^ T-cells (open circles). Blue symbols represent sequences that predict CCR5 coreceptor use; red symbols represent sequences that predict CXCR4 coreceptor use. The bar indicates genetic distance. Bootstrap values are expressed as percentages for each relevant node and represent the percentage occurrence of that node calculated using 100 bootstrap replicates. (A) Phylogenetic tree of HIV-1 *env* sequences of patient 18 (B) Phylogenetic tree of HIV-1 *env* sequences of patient 29.

Molecular *env* clones obtained from the monocytes of six patients were compared to the *env* clones obtained from CD4^+^ T-cells purified by negative magnetic selection from the same patients. Phylogenetic analysis revealed marked compartmentalization of the HIV-1 quasispecies between the monocytes and the CD4^+^ T-cells in all six patients (the phylogenetic tree of patients 18 and 29 are shown in Fig. 1A and 1B; Fig. S2 in the supplemental material gives the sequence alignments). HIV-1 was also compartmentalized between the CD14^high^ CD16^−^ and CD16^+^ monocytes in the patient whose HIV-1 DNA had been amplified from both monocyte subsets (Fig. 1B).

### Coreceptor usage of HIV-1 in monocytes from patients on effective cART

HIV-1 coreceptor usage was genotypically determined from the V3 amino-acid sequence of the *env* molecular clones obtained from the monocyte subsets of six patients; four in the CD14^high^ CD16^−^ subset, one in the CD16^+^ monocyte subset, and one in both monocyte subsets. CXCR4-using viruses were found in the CD14^high^ CD16^−^ monocytes of four (patients 15, 16, 18, 19) of five analyzable patients, whereas only CCR5-using viruses were found in the CD16^+^ monocytes of the two analyzable patients (patients 22 and 29). Two of the patients who harbored CXCR4-using viruses in their CD14^high^ CD16^−^ monocytes also harbored CXCR4-using viruses in their CD4^+^ T cells, albeit genetically distinct (patients 15 and 16), whereas the remaining two patients had only CCR5-using viruses in their CD4^+^ T-cells (patients 18 and 19). The V3 amino-acid sequences of the CXCR4-using viruses found in the CD14^high^ CD16^−^ monocytes revealed a QR insertion immediately before the GPGR crown in two patients, and a net charge of ≥+7 in three patients (see Fig. S2 in the supplemental material for sequence alignments).

### Residual plasma HIV-1 RNA below 50 copies/ml

The HIV-1 RNA in the plasma of most patients on cART becomes undetectable by standard assays with a sensitivity of 50 copies/ml. We therefore developed an ultrasensitive RT-PCR assay to measure HIV-1 RNA in the plasma at concentrations below 50 copies/ml. This assay reliably detected 2.5 copies/ml, as assessed using plasma samples containing known copy numbers of HIV-1 (NIBSC HIV-1 RNA 2nd international standard diluted in HIV-1-seronegative plasma). The reproducibility and linearity of the assay was validated in five independent experiments using serial dilutions of HIV-1 RNA. A plot of the measured values versus the input HIV-1 RNA was linear (R^2^ = 0.99) across a wide range of HIV RNA concentrations (see Fig. S3 in the supplemental material).

**Figure 2:**
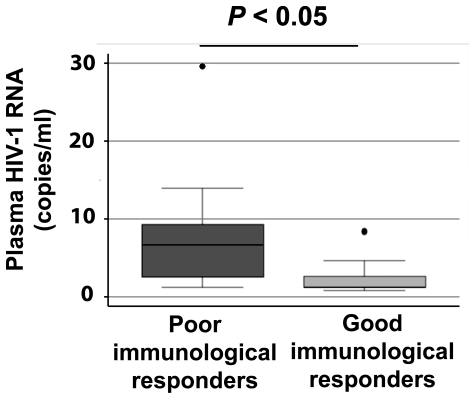
Residual HIV-1 RNA in the plasma. Comparison of the level of residual HIV-1 RNA in the plasma of the poor (n = 10) and good immunological responders to cART (n = 13).

We detected low-level viremia in 13 of the 23 patients, more frequently in the poor immunological responders (8/10 patients) than in the good immunological responders (5/13 patients) (*P* = 0.06). Samples with undetectable HIV-1 RNA were assigned a value half the detection threshold (1.2 copies/ml). The residual plasma virus load was higher in the poor immunological responders (median, 6.6 copies/ml, n = 10) than in the good immunological responders (median 1.2 copies/ml, n = 13) (*P*<0.05) (Fig. 2). The residual plasma virus load on cART was not significantly correlated with the plasma virus load before the initiation of cART, nor was it correlated with the nadir of CD4^+^ T-cells, and the CD4^+^ T-cell count on cART (*P*>0.10 for all). The residual HIV-1 RNA load in the plasma was also not correlated with the HIV-1 DNA load in the PBMCs on cART (ρ = −0.06, *P* = 0.79) (data not shown).

### Coreceptor usage of residual HIV-1 in the plasma on effective cART

We used a nested RT-PCR with a sensitivity of 2.5 copies/ml to amplify a region spanning the V3 *env* region and thus determine the coreceptor usage of the residual viruses in the plasma. HIV-1 V3 *env* was successfully amplified from the plasma of 12 of the 23 patients. The *env* PCR products were cloned and sequenced. CXCR4-using variants were found in the plasma of 5/12 patients (three poor and two good immunological responders).

### Phylogenetic relationships between the residual HIV-1 in the plasma and monocytes and CD4^+^ T-cells of patients on effective cART

We first assess the phylogenetic relationships between the sequences of the viruses present in the plasma, the monocytes, and the CD4^+^ T-cells during cART (see Fig. S2 in the supplemental material for sequence alignments).

**Figure 3:**
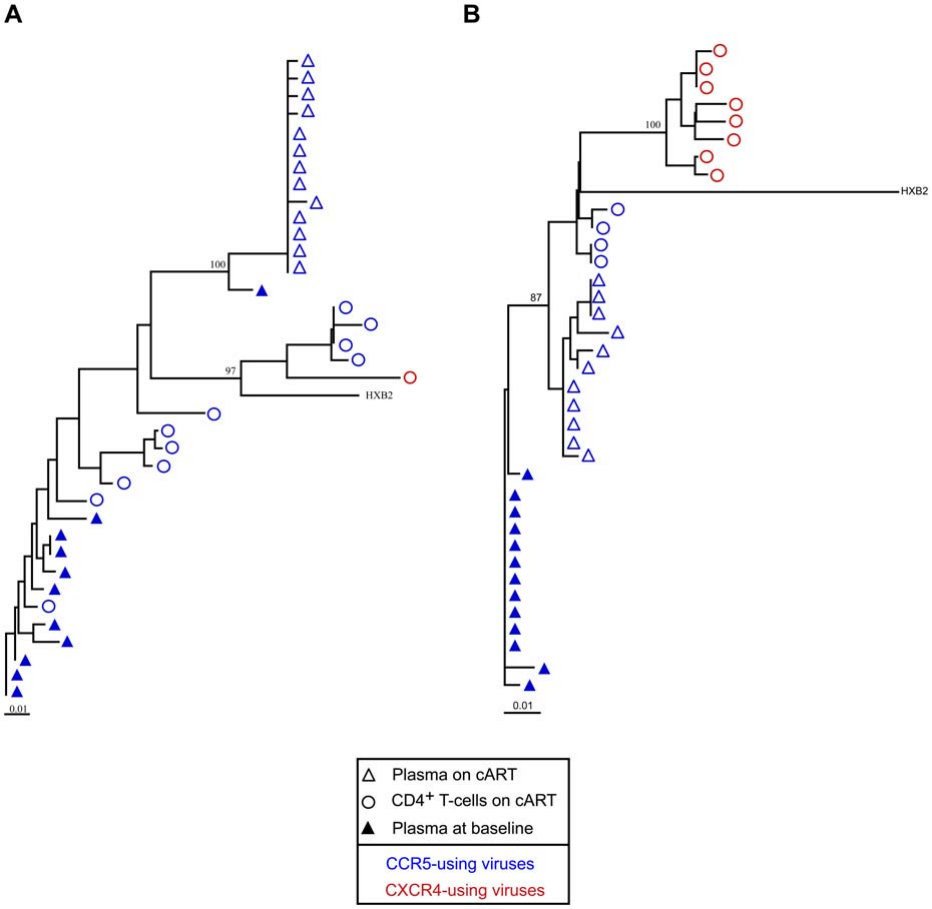
Difference between plasma and CD4^+^ T-cells viruses during cART and plasma viruses at baseline. Phylogenetic trees of HIV-1 *env* sequences obtained by clonal analysis of PCR products from CD4^+^ T-cells (open circles), plasma on cART (open triangles) and plasma at baseline (closed triangles). Blue symbols represent sequences that predict CCR5 coreceptor use; red symbols represent sequences that predict CXCR4 coreceptor use. The bar indicates genetic distance. Bootstrap values are expressed as percentages for each relevant node and represent the percentage occurrence of that node calculated using 100 bootstrap replicates. (A) Phylogenetic tree of HIV-1 *env* sequences of patient 2 (B) Phylogenetic tree of HIV-1 *env* sequences of patient 3.

**Figure 4:**
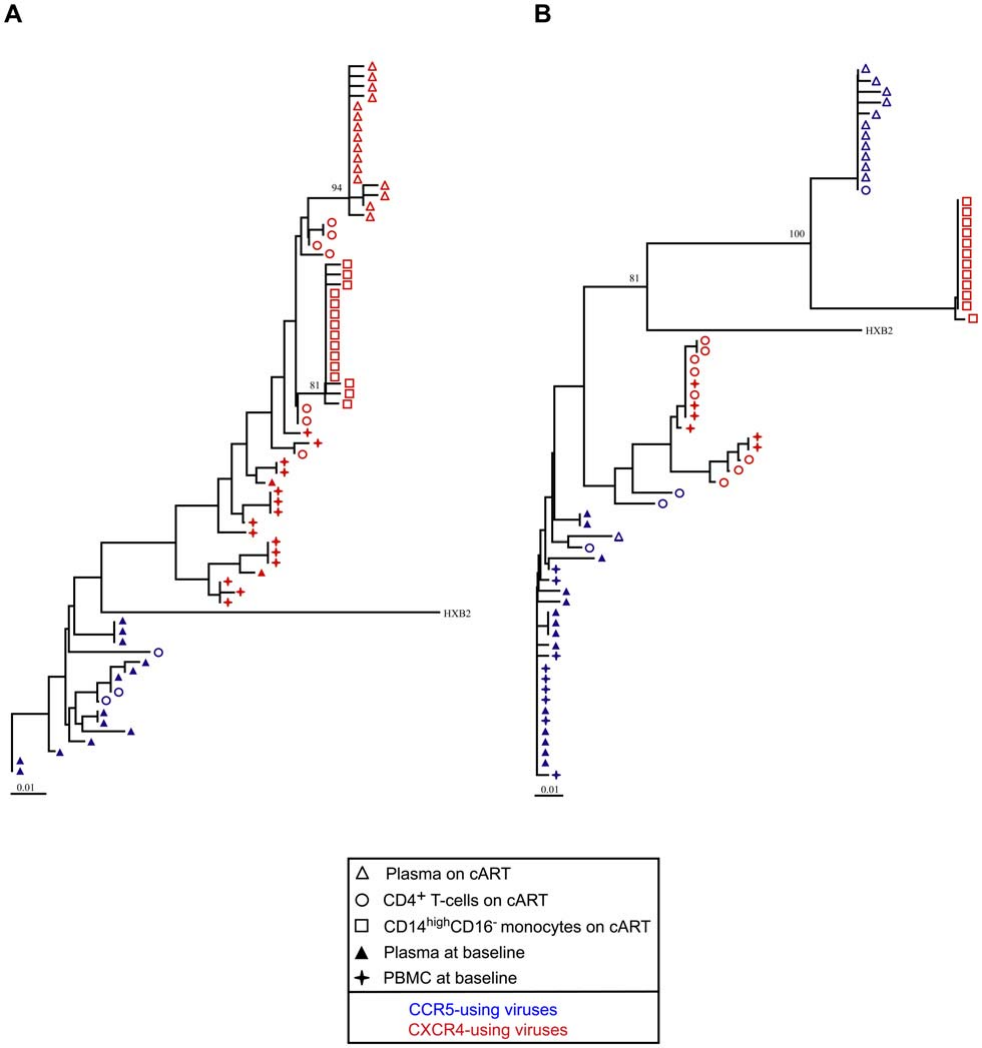
Genetic evolution of HIV-1 despite effective cART. Phylogenetic trees of HIV-1 *env* sequences obtained from CD14^high^ CD16^−^ monocytes (open squares), CD4^+^ T-cells (open circles), and plasma (open triangles) during cART, and plasma (closed triangles) and PBMCs (closed stars) at baseline before starting cART. Blue symbols represent sequences that predict CCR5 coreceptor use; red symbols represent sequences that predict CXCR4 coreceptor use. The bar indicates genetic distance. Bootstrap values are expressed as percentages for each relevant node and represent the percentage occurrence of that node calculated using 100 bootstrap replicates. (A) Phylogenetic tree of HIV-1 *env* sequences of patient 16 (B) Phylogenetic tree of HIV-1 *env* sequences of patient 15.

The sequences of the viruses in the plasma of four patients on cART appeared to be intermixed with the sequences found in the CD4^+^ T-cells (patients 1, 11, 20, 25). For three other patients (6, 15, 16), the sequences present in the plasma were closely related to some minor CD4^+^ clones. Finally, the sequences in the plasma of four patients (2, 3, 12, 29) were clearly distinct from all of the sequences found in their CD4^+^ T-cells, suggesting that these cells are not the source of the residual viremia in these patients (see patients 2 and 3 on Fig. 3). The residual sequences in the plasma on cART were also compared to those found in circulating monocytes at this time in three patients (15, 16, 29). Phylogenetic analysis revealed marked compartmentalization of the HIV-1 quasispecies between these two compartments (see patients 15 and 16 on Fig. 4).

### Genetic evolution between ancestral and recent virus sequences during cART

We next determined whether genetic evolution may have occurred from the ancestral virus sequences present at baseline in the plasma and/or the PBMCs to the sequences of recently produced viruses found in the plasma and the monocytes during cART. The sequences found in the baseline PBMCs of the nine patients for whom cell samples were available appeared to be intermixed with baseline plasma sequences. The residual *env* sequences in the plasma of six patients (1, 11, 12, 13, 20, 25) on cART appeared to be identical or very similar to pre-ART sequences. By contrast, the phylogenetic analyses suggest genetic evolution from ancestral to recent sequences during cART for the other six patients (2, 3, 6, 15, 16, and 29). PBMC samples taken at baseline were available for three of them (patients 15, 16, and 29). The HIV-1 *env* sequences found in the plasma and the monocytes of these patients after prolonged cART were clearly distinct from the sequences present in their plasma and cellular reservoirs at baseline (see patients 15 and 16 on Fig. 4).

### Impact of residual viremia on T-cell homeostasis of patients on cART

**Figure 5:**
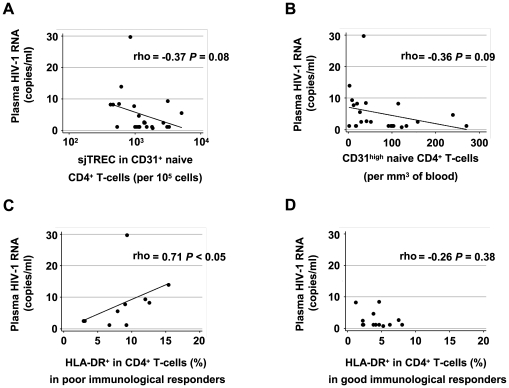
Correlation between residual viremia and immunological parameters of CD4^+^ T-cell homeostasis. (A) Correlation between the residual plasma virus concentration and the frequency of sjTREC-bearing cells in the CD31^+^ subset of naïve CD4^+^ T-cells; (B) Correlation between the residual plasma virus concentration and the absolute numbers of CD31^high^ naïve CD4^+^ T-cells per mm^3^ of blood; (C and D) Correlation between the residual plasma virus concentration and CD4^+^ T-cell activation in the poor and good immunological responders.

We finally assessed the impact of residual viremia on T cell homeostasis during cART. HIV-1 replication can interfere with the production of new naïve T cells by the thymus [54]. The thymus could also be a site of virus persistence in patients on cART, and hence a source of residual virus [55], [56]. We therefore assessed the relationship between thymus function, previously assessed in the patients of the ANRS EP32 study, and the residual plasma HIV-1. The residual viremia was not significantly correlated with the intrathymus proliferation rate of immature thymocytes, as assessed by measuring the sj/βTREC ratio in PBMCs (ρ = −0.00, *P* = 0.99). But the residual viremia tended to be negatively correlated with the frequency of recent thymic emigrants in CD31^+^ naive CD4^+^-T cells, measured by the frequency of sjTREC-bearing cells in the CD31^+^ naïve-cell subset (ρ = −0.37, *P* = 0.08) (Fig. 5A). There was a similar tendency to a negative correlation with the frequency of sjTREC-bearing cells in the naïve CD8^+^ T-cell subset (ρ = −0.41, *P* = 0.05, data not shown). Finally, the residual viremia also tended to be negatively correlated with the absolute number of recent thymic emigrants per mm^3^ of blood, defined by a CD31^high^ phenotype among the naïve CD4^+^ T-cells (ρ = −0.36, *P* = 0.09) (Fig. 5B). This tendency to fewer recent thymic emigrants in the peripheral blood of those patients who have the highest residual viremia was not linked to HIV-1 coreceptor usage, nor did it differ between the poor and good immunological responders (data not shown). Furthermore, there were significant genetic differences between the residual virus sequences found in the plasma and the sequences found in the CD31^+^ naïve CD4^+^ T-cells during cART (data not shown). These results, therefore, do not indicate that the thymus is a source of the residual viruses found in the plasma.

We have previously shown that poor immune reconstitution of patients on cART is strongly correlated with persistent T-cell activation rather than with thymus dysfunction [28]. Residual virus replication despite cART could be one of the drivers of persistent T-cell activation. We therefore assessed the association between residual viremia and persistent T-cell activation in patients on cART with a sustained plasma virus load of <50 copies/ml. The level of residual viremia was correlated with the frequency of HLA-DR^+^ CD4^+^ T-cells (ρ = 0.41, *P* = 0.05) and of HLA-DR^+^ CD38^+^ CD8^+^ T-cells (ρ = 0.40, *P* = 0.06) (data not shown). As the residual viremia was greater in the poor immunological responders than it was in the good immunological responders, we then investigated the relationship between residual viremia and T-cell activation in the two groups separately. The level of residual viremia was significantly correlated with the frequency of activated CD4^+^ T-cells (ρ = 0.71, *P*<0.05) in the poor immunological responders (Fig. 5C), but not in the good immunological responders (ρ = -0.26, *P* = 0.38) (Fig. 5D). Similarly, the level of residual viremia was significantly correlated with the frequency of activated CD8^+^ T-cells in the poor immunological responders (ρ = 0.71, *P*<0.05), but not in the good immunological responders (ρ = −0.12, *P* = 0.70) (data not shown). These results suggest that low-level HIV-1 viremia may contribute to persistent T-cell activation and poor immune reconstitution despite cART.

## Discussion

We have investigated the residual replication of HIV-1 in 23 patients that were receiving sustained effective cART for a median duration of seven years. Residual plasma virus below 50 copies per ml was detected in 8 of 10 patients with poor CD4^+^ T-cell reconstitution in response to cART, and in only 5 of 13 patients with good CD4^+^ T-cell reconstitution. This confirms previous reports [1]–[7] that low-level virus production can continue in patients despite their being on long-term effective cART. Our results further suggest that this residual viremia could have a significant impact on the immunological parameters of some patients on sustained effective cART.

One important issue for assessing virus populations based on PCR products is the use of a sufficient number of templates. To limit resampling bias in this study, we ultracentrifuged 6 ml of plasma to characterize the residual plasma viremia. As the levels of residual HIV-1 RNA detected in the plasma ranged from 2.5 to 29.7 copies/ml,we can thus estimate that 15 to 180 copies of HIV-1 were subjected to V3 *env *PCR in these patients. Moreover, there was significant genetic diversity in the residual viruses found in the plasma of some patients, despite their residual viremia being rather low. In contrast, there was little genetic diversity in the residual viruses found in the plasma of most patients despite their higher residual viremia. This finding of a homogenous virus population in the plasma of most of the patients on effective cART is in agreement with other reports. In a study designed to avoid PCR resampling using intensive sampling, Bailey *et al*. found a predominant plasma clone in most of the patients with treatment-induced viral loads below 50 copies/ml [1]. Indeed, despite wide diversity of HIV-1 sequences in resting CD4^+^ T-cells, they found that residual virus in plasma was dominated by a homogeneous population with identical *pol* and *env *sequences.

We investigated the cellular sources of the low-level viremia that persists during cART and found that the residual viruses in the plasma of most patients on long-term effective cART were phylogenetically similar to the viruses in their CD4^+^ T-cells, the major cellular reservoir of HIV-1. But, in agreement with others [1], [57], our results also suggest that the CD4^+^ T-cells are probably not the only source of the residual viruses in the plasma of some patients. Several reports have suggested that blood monocytes are one of the sources of the viruses in the plasma of patients on cART [32], [57]. However, we find that the viruses in the plasma and monocytes on cART are clearly different, suggesting that these cells were not the source of the residual plasma viruses. As both compartments are probably replenished by recently produced viruses, there could be at least two sources of residual virus in patients on effective cART. Circulating monocytes are thought to reflect recent infection because of their short half-life in the peripheral blood [36], [37]. But a stable latent infection of some of their bone marrow progenitors might challenge this view. However, despite some controversy, hematopoietic CD34^+^ stem cells do not appear to be frequently infected by HIV-1 *in vivo* [58]–[60]. By contrast, HIV-1 could infect further committed monocytic progenitors and contribute to the presence of HIV-1 DNA in circulating monocytes (reviewed in [61]). These committed hematopoietic progenitors have lost their self-renewal capacity and have a short half-life before terminally differentiating into blood monocytes [36], [37]. Hence, they cannot be a stable latent reservoir for HIV-1. While we cannot determine whether circulating monocytes have been infected in the bone marrow or after their release in the peripheral blood, they probably reflect recently produced viruses. A longitudinal study reported that the virus sequences found in blood monocytes continuously evolve faster during cART than the virus sequences found in the CD4^+^ T cell compartment, which is in favor of the labile status of the viruses found in circulating monocytes [34].

Low-level viremia could arise from the release of archival virus from latent cellular reservoirs established before cART or from low-levels of complete cycles of virus replication. Whether ongoing low-level virus replication may persist despite apparently effective cART remains controversial, as previous studies have obtained conflicting results. The residual plasma HIV-1 RNA load has been reported to decrease following treatment intensification with abacavir [3]. Similarly, a transient increase in unintegrated episomal forms of cell-associated HIV-1 DNA was observed following treatment intensification with raltegravir [62]. However, studies suggesting that virus replication may persist despite cART are challenged by others that found no effect of treatment intensification on the residual plasma HIV-1 RNA load [63]–[66].

Our results suggest that both viral expression from cellular reservoirs and ongoing viral replication may occur on cART. Half of the patients in our study showed no significant difference between the ancestral viruses found in their plasma and PBMCs at baseline before starting cART and the residual viruses in their plasma after they had been on cART. These findings are thus compatible with the sporadic release of ancestral viruses archived in cellular reservoirs into the plasma without any significant genetic evolution in these patients during cART, in agreement with previous reports [15]–[17], [66], [67]. By contrast, phylogenetic analyses suggest that there was genetic evolution of the virus sequences in six patients, despite their being on cART. The sequences of ancestral viruses in baseline plasma and PBMCs had evolved to those of recent viruses found in the plasma and monocytes of these patients on cART. The genetic evolution from ancestral to recent sequences in these patients suggests that there can still be cycles of virus replication in some patients despite apparently effective cART.

While the absence of significant residual virus replication and genetic evolution during cART is probably the most frequent pattern, the ongoing virus replication despite cART suggested by our results might be due to some particular characteristics of the subjects studied here. They are not representative of the general population of HIV-infected individuals, but were selected because of their markedly different phenotypes of poor and good immunological reconstitution in response to cART. They also have different virological characteristics, with CXCR4-using viruses being frequent in the poor immunological responders [28]. All the patients in this study with a pattern suggesting ongoing virus replication and genetic evolution during cART harbored CXCR4-using viruses. This suggests a link between ongoing virus replication during cART and virus tropism for CXCR4.

We previously reported that cART seems to favor the progressive selection of pre-existing minor CXCR4-viruses that were already present in baseline quasispecies [43]. Other studies have also reported an increased frequency of CXCR4-using viruses in patients on cART [68]-[70]. How cART might favor the residual replication of CXCR4-using viruses remains unknown. One study reported that the expression of CXCR4 was correlated with the presence of P-glycoprotein in CD4^+^ T lymphocytes [71]. CXCR4-bearing cells could thus be a pharmacological sanctuary. Alternatively, the lymphoid microenvironment could provide a selective advantage for the replication of CXCR4-using viruses during cART, in parallel with changes in T-cell homeostasis during immune reconstitution.

Our results also suggest that the CD14^high^ CD16^−^ monocytes could play a role in the emergence of CXCR4-using viruses during cART. We found an unexpectedly high frequency of CXCR4-using viruses in the CD14^high^ CD16^−^ monocytes, even in some patients who had only CCR5-using viruses in their CD4^+^ T-cells. By contrast, all the molecular *env* clones from CD16^+^ monocytes were predicted to use CCR5. This could be due to different levels of expression of the HIV-1 entry coreceptor on the surfaces of these two monocyte subsets. CD16^+^ monocytes bear two to five times more CCR5 and two to four times less CXCR4 than do CD14^high^ CD16^−^ monocytes [51].

The residual replication of CXCR4-using viruses could interfere with both thymus activity and peripheral T-cell homeostasis as thymocytes and naïve CD4^+^ T-cells mainly bear the CXCR4 coreceptor for HIV-1 [44], [55], [72]. We find a tendency to fewer recent thymic emigrants in the peripheral blood of those patients with the highest residual viremia. HIV-1 replication can interfere with the production of new naïve T-cells by the thymus, either directly by infecting immature or mature CD4^+^ thymocytes [55], or indirectly by reducing the proliferation of T-cell precursors within the thymus, due to inhibition by certain cytokines, notably interferon-α [73]. However, the ANRS EP32 study showed that the infection of CD4^+^ thymocytes and their export into the peripheral circulation is probably rare in patients on effective cART. Most of the infectious events in naïve CD4^+^ T-cells seem to occur during the peripheral proliferation of these cells, as suggested by the finding of more virus in the CD31^−^ naïve CD4^+^ T-cells that have proliferated in the periphery than in the CD31^+^ subset that contains the recent thymic emigrants [28]. We found that the sequences of the viruses in the CD31^+^ naïve CD4^+^ T-cells were different from those in the plasma, suggesting that the thymus is probably not a source of the residual virus in the plasma of patients on cART. Furthermore, our data do not support the idea that the residual viremia inhibits thymus output via a cytokine pathway. We found no negative correlation between the residual viremia and the intrathymic proliferation of immature thymocytes, measured by the sj/βTREC ratio. Thus, the residual viremia does not seem to inhibit thymus output, either directly or indirectly. But the tendency for there to be fewer recent thymic emigrants in the peripheral blood of those patients with the highest residual viremia might be best explained by their sequestration in secondary lymphoid organs, as in viremic patients [74], [75]. Unfortunately, biopsies of the lymph nodes of the patients in the ANRS EP32 study were not available to confirm this hypothesis.

Our previous study comparing the virological and immunological features of these patients showed that poor immunological reconstitution despite effective cART was strongly correlated with persistent T-cell activation rather than with thymus disfuction [28]. The reports on the capacity of residual virus replication despite cART to drive persistent T-cell activation are conflicting. The presence of the CD38 marker of activation on CD8^+^ T-cells is correlated with the HIV-1 RNA load in the plasma and declines during cART. It has thus been suggested that the persistence of increased frequencies of CD38^high^ CD8^+^ T-cells in patients on effective cART reflects residual virus replication [76], [77]. One study [3] showed that some markers of cell activation were reduced in both CD4^+^ and CD8^+^ T-cells following intensified antiretroviral treatment. The residual viremia was also associated with immune activation, as indicated by soluble blood markers but not by cellular markers of immune activation [78]. By contrast, another recent study found no association between the percentage of CD38^high^ CD8^+^ T-cells and the residual plasma virus, although CD8^+^ T-cell activation was negatively correlated with the CD4^+^ T-cell count [79]. Our data indicate that there is more residual HIV-1 RNA in the plasma of poor immunological responders than in that of good immunological responders. We also find that the residual viremia in the poor immunological responders was positively correlated with the activation of their CD4^+^ and CD8^+^ T-cells, as assessed by the HLA-DR and CD38 markers. The ongoing low-level virus production despite cART in some patients might thus contribute to persistent immune activation.

We have characterized the residual viruses that are still produced in HIV-infected patients despite their being on sustained effective cART for a median duration of seven years. We find evidence that the residual viremia could be due to the release of archival virus from reservoir cells and/or ongoing virus replication in some patients. The compartmentalization of recently produced viruses between the plasma and the blood monocytes suggests that there are at least two sources of the residual virus found in patients on effective cART. Moreover, other cellular sources of viruses, in addition to the main CD4^+^ T-cell reservoir, could also contribute to the plasma residual viruses. CXCR4-using viruses were frequent among the recently produced viruses in the plasma and in the main CD14^high^ CD16^−^ monocyte subset, suggesting that these variants might be produced preferentially in patients on cART. Finally, our data strongly suggest that low-level virus production might contribute to persistent immune dysfunction in some patients, despite cART.

## Supporting Information

Table S1 Clinical and biological patient characteristics.
(0.07 MB PDF)

Figure S1 Isolation of CD14high CD16- and CD16+ monocyte subsets by flow cytometry (flow cytometry of patient 10 is shown). Blood monocytes were sorted from cryopreserved PBMCs. Monocytes were defined with forward and side scatter, and were then sorted based on CD14 and CD16 expression among CD3- CD4low CD56- cells.
(0.06 MB DOC)

Figure S2 V3 env sequences alignments and predicted coreceptor use of HIV-1. HIV-1 sequences found at baseline and during effective cART are shown for patients in whom viruses were detected in the plasma and/or monocytes during cART.
(0.11 MB PDF)

Figure S3 Linearity of the ultrasensitive plasma HIV-1 RNA assay. Plot of the measured values against the input template (copies per ml).
(0.07 MB PDF)
