# Supplementary material for: HIV-1 Residual Viremia Correlates with Persistent T-Cell Activation in Poor Immunological Responders to Combination Antiretroviral Therapy
Source: PLoS One. 2009 Oct 30;4(10):e7658. doi: 10.1371/journal.pone.0007658 (PMC2765414; doi:10.1371/journal.pone.0007658)
Supplement: Table S1 — Clinical and biological patient characteristics. (0.07 MB PDF) [file pone.0007658.s001.pdf]

Immune reconstitution group	Patient n°	Gender	Age[a]	Duration of HIV-1 infection since diagnosis[a]	$CD4^+$ T-cell count nadir[b]	Pre-cART $CD4^+$ T-cell count[b]	Pre-cART plasma viral load[c]	Duration of cART[d]	cART regimen at time of analysis	$CD4^+$ T-cell count at time of analysis[b]	Plasma viral load at time of analysis[e]
**Poor immunological responders**	1	M	41	13	91	91	5	65	3TC EFV RTV/FPV	268	<50
	2	M	47	14	66	66	5	25	AZT 3TC LPV/RTV	164	<50
	3	M	43	8	71	71	2.9	97	AZT 3TC ABC	236	<50
	6	M	34	3	62	62	4.6	36	AZT 3TC EFV	229	<50
	8	M	62	12	4	4	5.9	98	AZT 3TC NVP	179	<50
	10	M	65	11	5	22	4	98	3TC ABC NVP	239	<50
	11	M	54	12	51	51	4.6	60	AZT 3TC ABC	278	<50
	12	M	57	13	106	211	6.6	36	AZT 3TC LPV/RTV	233	<50
	13	M	58	6	75	101	4.8	84	AZT 3TC NVP	219	<50
	15	M	58	16	7	114	6	64	AZT 3TC ABC EFV	206	<50
**Good immunological responders**	16	F	50	15	155	155	6.1	94	3TC TDF NVP	985	<50
	17	M	46	10	195	195	4.1	84	DDI FTC EFV	771	<50
	18	M	47	8	22	139	5.7	48	AZT 3TC ABC	776	<50
	19	M	42	6	156	323	4.6	72	AZT 3TC ABC	716	<50
	20	M	48	7	111	111	5.5	72	D4T 3TC IDV	818	<50
	22	M	42	14	66	66	5.5	84	AZT 3TC NVP	888	<50
	24	M	51	8	281	281	3.9	48	AZT 3TC ABC	730	<50
	25	F	63	8	205	205	4.9	96	3TC ABC NVP NFV	910	<50
	26	M	47	7	167	167	5.6	84	D4T 3TC IDV	813	<50
	27	F	40	10	197	220	5.2	104	D4T 3TC NVP	757	<50
	28	M	41	11	1	1	6.3	72	D4T 3TC IDV/RTV	1132	<50
	29	M	36	9	102	102	5.8	91	D4T 3TC NVP	790	<50
	30	M	38	14	112	112	5.2	84	AZT 3TC NFV	987	<50
***P* between groups**	-	0.23	0.11	0.40	<0.01	0.02	0.42	0.32	-	-	-

[a] years [b] cells/mm^3 [c] log_{10} copies/ml [d] months [e] copies/ml
