# Supplementary figures and images for: HIV-1 Residual Viremia Correlates with Persistent T-Cell Activation in Poor Immunological Responders to Combination Antiretroviral Therapy
Source: PLoS One. 2009 Oct 30;4(10):e7658. doi: 10.1371/journal.pone.0007658 (PMC2765414; doi:10.1371/journal.pone.0007658)

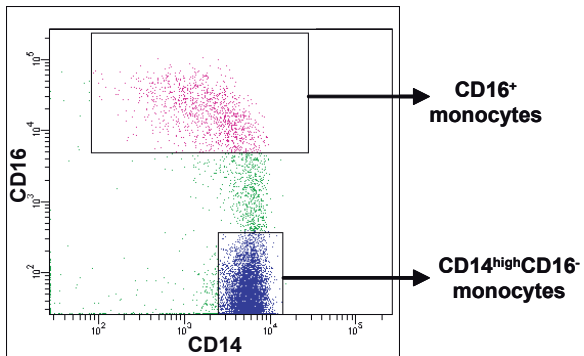

Supplement: Figure S1 — Isolation of CD14high CD16- and CD16+ monocyte subsets by flow cytometry (flow cytometry of patient 10 is shown). Blood monocytes were sorted from cryopreserved PBMCs. Monocytes were defined with forward and side scatter, and were then sorted based on CD14 and CD16 expression among CD3- CD4low CD56- cells. (0.06 MB DOC) [file pone.0007658.s002.pdf]

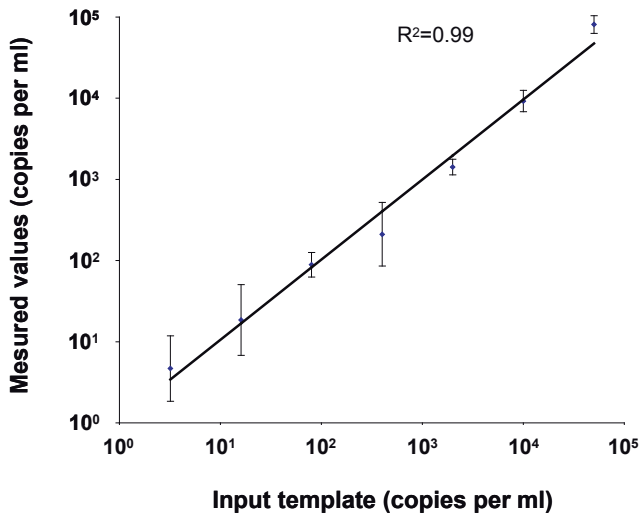

Supplement: Figure S3 — Linearity of the ultrasensitive plasma HIV-1 RNA assay. Plot of the measured values against the input template (copies per ml). (0.07 MB PDF) [file pone.0007658.s004.pdf]
