# Supplementary material for: HIV-1 Residual Viremia Correlates with Persistent T-Cell Activation in Poor Immunological Responders to Combination Antiretroviral Therapy
Source: PLoS One. 2009 Oct 30;4(10):e7658. doi: 10.1371/journal.pone.0007658 (PMC2765414; doi:10.1371/journal.pone.0007658)
Supplement: Figure S2 — V3 env sequences alignments and predicted coreceptor use of HIV-1. HIV-1 sequences found at baseline and during effective cART are shown for patients in whom viruses were detected in the plasma and/or monocytes during cART. (0.11 MB PDF) [file pone.0007658.s003.pdf]

Patient no.	No. of clones / total tested	Amino acid sequence of the V3 *env* region	Predicted genotype
**1**			
	**Plasma at baseline**		
	Consensus	**C T R P N N N T R K G I R V G P G R A I Y A T E K I I G N I R Q A H C**	
	6/14	. . . .	**X4**
	1/14	. . . . **E**	**X4**
	1/14	. . . **H**	**X4**
	2/14	. . . **H** . . . . . . **S** . **H I** . . . . . **F** . . . **G E** . **V** . **D**	**R5**
	1/14	. . . **H** . . . . . . **S** . **H I** . . . . . **F** . . . **G E M V** . **D**	**R5**
	1/14	. . . . **S** . **H I** . . . . . **F** . . . **G D** . . . **D**	**R5**
	1/14	. . . **H** . . . . . . **S** . **H I** . . . . . **F** . . . **G E** . . . **D**	**R5**
	1/14	. . . **H** . . . . . . **S** . **H I** . . . . . **F** . . . **G D** . . . **D**	**R5**
	**Plasma on cART**		
	Consensus	**C T R P N N N T R K G I R I G P G R A I Y A T E K I I G N I R Q A H C**	
	8/23	. . . . **V**	**X4**
	1/23	. . . . **V** . . . . **T**	**X4**
	1/23	**R** . . . . **V**	**X4**
	6/23	. . . .	**X4**
	1/23	. . . . **A**	**X4**
	4/23	. . . **H** . . . . . . **S** . **H** . . . . . . **F** . . . **G E** . . . **D**	**R5**
	1/23	. . . . **S** . **H** . . . . . . **F** . . . **G G** . . . **D**	**R5**
	1/23	. . . . **S** . **H** . . . . . . **F** . . **A G G** . . . **D**	**R5**
	**CD4⁺ T-cells on cART**		
	Consensus	**C T R P N N N T R K G I R V G P G R A I Y A T E K I I G N I R Q A H C**	
	8/12	. . . .	**X4**
	1/12	. . . . **L**	**X4**
	1/12	. . . . **I**	**X4**
	1/12	. . . . **K** . . . . . **R** . . . . **K**	**X4**
	1/12	. . . **H** . . . . . . **S** . **H I** . . . . . **F** . . . **G G** . . . **D**	**R5**
**2**			
	**Plasma at baseline**		
	Consensus	**C T R P N N N T R K S I N I A P G R A I Y A T G A I I G N I R Q A H C**	
	8/11	. . . .	**R5**
	2/11	. . . . **D**	**R5**
	1/11	. . . . **G** . **H M G** . . **G T F F**	**R5**
	**Plasma on cART**		
	Consensus	**C T R P N N N T R K G I H M G P G G T F F A T G A I I G N I R Q A H C**	
	10/13	. . . .	**R5**
	1/13	. . . . **V**	**R5**
	1/13	. . . . **V**	**R5**
	1/13	. . . . **L**	**R5**
	**CD4⁺ T-cells on cART**		
	Consensus	**C T R P N N N T R K S I H I G P G R A F Y A T G Q I I G D I R Q A H C**	
	4/12	. . . . **G** . . **M** . . . **~** . . . **T**	**R5**
	3/12	. . . . **T** . . . . . **S** . **F** . . . **G**	**R5**
	1/12	. . . . **T** . . . . . **S** . **F** . . . **G** . . . **N**	**R5**
	2/12	. . . . **N** . **A** . . . . **I** . . . . **A** . . . **N**	**R5**
	1/12	. . . . **P** . . . . **D**	**R5**
	1/12	. . . . **G** . **R** . . . . **I T** . **D R**	**X4**
**3**			
	**Plasma at baseline**		
	Consensus	**C T R P N N N T R K ~ G I H I G P G R A F Y T T G Q I I G D I R Q A H C**	
	12/13	. . . . **~**	**R5**
	1/13	. . . . **S** . . . . . **~**	**R5**
	**Plasma on cART**		
	Consensus	**C T R P N N N T R K ~ S I H I G P G S A F Y T T G Q I I G D I R Q A H C**	
	8/11	. . . . **~**	**R5**
	1/11	. . . . . . **I** . . . **~**	**R5**
	1/11	. . . . **D** . . . . . **~**	**R5**
	1/11	. **A** . . . . **~**	**R5**
	**CD4⁺ T-cells on cART**		
	Consensus	**C T R P N N N I K K R R I H I G P G R A F Y T T G Q L I G D I R Q A H C**	
	2/12	. . . . **H** . **S** . . **R** . . . . **~**	**X4**
	1/12	. . . . **H** . **S** . . . . **R** . . **~**	**X4**
	2/12	. . . . **R** . . **~**	**X4**
	3/12	. . . . . **S** . . . . **R** . . **~**	**X4**
	3/12	. . . . **T R** . **~ S** . . . . **I**	**R5**
	1/12	. . . . **T R** . **~ S** . . **T** . . . . **I**	**R5**
**6**			
	**Plasma at baseline**		
	Consensus	**C T R P N N N T R K G I H V G P G R T L Y A T G D I T G D I R Q A H C**	
	6/10	. . . .	**R5**
	1/10	. . . . **I**	**R5**
	1/10	. . . . **M** . . . . . **V** . . . . **E** . **I**	**R5**
	1/10	. . . . **M** . . . . . **V** . . . . . . **I**	**R5**
	1/10	. . . . **T** . . . . **E** . . . . **R** .	**R5**

Patient no.	No. of clones / total tested	Amino acid sequence of the V3 *env* region	Predicted genotype
**6 (continued)**			
**Plasma on cART**			
	Consensus	**C T R P N N N T R R G I R I G P G R T L Y A T Q K I I G D I R Q A H C**	
	12/15	. . . .	**X4**
	1/15	. . . . S	**X4**
	1/15	. . . . R	**X4**
	1/15	R	**X4**
**$CD4^+$ T-cells on cART**			
	Consensus	**C T R P N N N T R R G I H I G P G R T L Y A T Q K I I G D I R Q A H C**	
	3/11	. . . . R	**X4**
	1/11	. . . .	**X4**
	2/11	. . . . S . . . . . K	**X4**
	2/11	. . . . R	**X4**
	1/11	. . S . . . . R	**R5**
	2/11	. . . . S . . . . A . . . . G D	**R5**
**11**			
**Plasma at baseline**			
	Consensus	**C E R P N N N T S K S I T M G P G R A F F V T G G I I G D I R Q A H C**	
	6/10	. . . .	**R5**
	1/10	. . . . I . . . . A . E R . . . . . . R . . .	**R5**
	2/10	. A . . G . . . . P L . . . . A . E R . . . . . . R . . .	**R5**
	1/10	. A . . G . . . . P L . . . . V . . A . E R . . . . . . R . . .	**R5**
**PBMC at baseline**			
	Consensus	**C E R P N N N T S K S I T M G P G R A F F V T G G I I G D I R Q A H C**	
	5/12	. . . .	**R5**
	1/12	. . . . S	**R5**
	2/12	. . . . L	**R5**
	1/12	. . . . I . . . . A . . R . . . N	**R5**
	1/12	. T . . . . . . R . . . . I . . . . A . . E	**R5**
	1/12	. . . . S . . . R . . . . I . . . . A	**R5**
	1/12	. V . . G . . . . P L . . . . A . E R	**R5**
**Plasma on cART**			
	Consensus	**C T R P G N N T S K S I P L G P G R A F F A T E R I I G D I R R A H C**	
	13/23	. . . .	**R5**
	1/23	. . . . T	**R5**
	1/23	. . . . T	**R5**
	2/23	. E . . N . . . . T M . . . . V . G . . . . N . . Q . . .	**R5**
	2/23	. E . . N . . . . T M . . . . V . G . . V . . . . Q . . .	**R5**
	2/23	. E . . N . . . . T I . . . . G . . . . Q . Y .	**R5**
	1/23	. E . . N . . . . T I . . . . G . . . R . . . Q . Y .	**R5**
	1/23	. E . . N . . . . T I . . . . G . . . . N . . Q . . .	**R5**
**$CD4^+$ T-cells on cART**			
	Consensus	**C E R P N N N T S K S I T M G P G R A F F V T G R I I G A I R Q A H C**	
	6/13	. . . .	**R5**
	1/13	. . . . . D . . . . M	**R5**
	5/13	. T . . G . . . . P L . . . . A . E . . . . D . . R . . .	**R5**
	1/13	. T . . G . . . . P L . . . . A	**R5**
**12**			
**Plasma at baseline**			
	Consensus	**C T R P S N N T R R G I H I G P G R A F Y A T G E V T G D I R Q A H C**	
	4/12	. . . .	**R5**
	4/12	. . . . T	**R5**
	1/12	. . . . Y . . . . K . . . . T . . T	**R5**
	2/12	. . . . G . . . . K . . Y . . . . . . V . . . E R I . . . . . R . . .	**X4**
	1/12	. . . . G . . . . K . . Y . . . . . . V . . . E R I . . . . . R . Y .	**X4**
**PBMC at baseline**			
	Consensus	**C T R P G N N T R K G I Y I G P G R A V Y A T E R I T G D I R R A H C**	
	10/14	. . . .	**X4**
	1/14	. . . . S . . . . R . . H . . . . . . F . . . G E V . . . . . Q . . .	**R5**
	1/14	. . . . S . . . . R . . H . . . . . . F . T . G E V . . . . . Q . . .	**R5**
	1/14	. . . . S . . . . R . . H . . . . G . F . T . G E V . . . . . Q . . .	**R5**
	1/14	. . . . S . S . . R . . H . . . . . . F . T . G E V . . . . . Q . . .	**R5**
**Plasma on cART**			
	Consensus	**C T R P S N N T R R G I H I G P G R A F Y A T G E V T G D I R Q A H C**	
	14/14	. . . .	**R5**
**$CD4^+$ T-cells on cART**			
	Consensus	**C T R P G N N T R K G I Y I G P G R A V Y A T E R I T G D I R R A H C**	
	6/10	. . . .	**X4**
	1/10	. . . . S . . . . T	**X4**
	1/10	. . . . S . . . . . S . . . . V I	**X4**
	1/10	. . . . Q . . .	**R5**
	1/10	. . . . Y . . . . H . . . . . T F . T . G E V . . . . . Q . . .	**R5**

Patient no.	No. of clones / total tested	Amino acid sequence of the V3 *env* region	Predicted genotype
**13**			
	**Plasma at baseline**		
	Consensus	**C I R P N N N T R K G I P M G P G K T L Y A A G E I I G N T R Q A H C**	
	3/14	. . . .	R5
	2/14	. . . . A	R5
	1/14	. . . . R	R5
	1/14	. . . . I	R5
	1/14	. . . . Q	R5
	1/14	. . . . Q . . . . R . . Y	R5
	1/14	. . . . Q . . . . . V . . . . K . . .	R5
	1/14	. . . . G . . . Q . . . . . V . . . . K . . .	R5
	1/14	. . . . Q . . . . . I . . . T . D	R5
	1/14	. T . . S . . . . H . . . . R . . . . T . A . T . D I	R5
	1/14	. T . . S . . . . T . . H . . . . R . . . . T . A . T . . I . K . Y .	R5
	**PBMC at baseline**		
	Consensus	**C T R P S N N T R K G I H M G P G R T L Y A T G A I T G N I R K A H C**	
	1/15	. . . . T . . . . Y .	R5
	1/15	. . . . D	R5
	1/15	. . . . R . . . . D . . Q . . .	R5
	5/15	. I . . N . . . . I . . E . . . . . . R D . I R . T . Q . . .	R5
	1/15	. I . . N . . . . Q . . . . K I . . . . . D . I . . T	R5
	1/15	. I . . N . . . . Q . . . . K . . . . A . E . I . . T . Q . . .	R5
	4/15	. I . . N . . . . P . . . . K . . . . . . E . I . . T . Q . . .	R5
	**Plasma on cART**		
	Consensus	**C T R P S N N T R K G I H M G P G R T L Y A T G A I T G N I R K A H C**	
	5/11	. . . .	X4
	1/11	. . . . R	X4
	5/11	. . . . D	R5
**15**			
	**Plasma at baseline**		
	Consensus	**C T R P G N N T K R S I T I G P G R A F Y T T E N I I G N I R Q A H C**	
	6/14	. . . .	R5
	3/14	. . . . A	R5
	3/14	. . . . A	R5
	1/14	. . . . E	R5
	1/14	. . . . K	R5
	**PBMC at baseline**		
	Consensus	**C T R P G N N T K R S I T I G P G R A F Y T T E N I I G N I R Q A H C**	
	9/15	. . . .	R5
	4/15	. . . . . . K . . . R M . . . . . K V . . . . ~ . . V	X4
	2/15	. . . . . . K . I K R M . . . . . K V . . . . ~ . . P	X4
	**Plasma on cART**		
	Consensus	**C E R P N N N T R K G I H M G P G K T L Y A T E N I I G N I R Q A H C**	
	11/12	. . . .	R5
	1/12	. T . . G . . . K . S . T I . . . R A F . . S	R5
	**$CD4^{+}$ T-cells on cART**		
	Consensus	**C T R P G N K T K R R M T I G P G K V F Y T T ~ N I V G N I R Q A H C**	
	4/12	. . . . ~	X4
	2/12	. . . . ~ . . . . Y .	X4
	1/12	. . . . I . . . . ~ . . P	X4
	1/12	. . . . ~ . . P	X4
	1/12	. . . . S I . . . . . R A . . . . ~ D . I	R5
	1/12	. . . . . . N . . . S I . . . . . R A . . . . ~ D . I . D	R5
	1/12	. . . . . . N . . K S I . . . . . R A . . . . E . . I	R5
	1/12	. E . . N . N . R K G I H M . . . . T L . A . E . . I	R5
	**$CD14^{high}$ $CD16^{-}$ monocytes on cART**		
	Consensus	**C A R P N N N T R K S I H I R P K K T L Y A T E D I I R K I R Q A H C**	
	11/12	. . . .	X4
	1/12	. . . . G	X4
**16**			
	**Plasma at baseline**		
	Consensus	**C T R P N N N T R ~ K S I H I G P G R A F F A T G Q I I G K I R E A H C**	
	3/15	. . . . ~	R5
	3/15	. . . . ~ . . . . Y	R5
	1/15	. . . . ~ . . . . S	R5
	2/15	. . . . ~ . . . . D	R5
	1/15	. . . . . . D . . ~ . . . . D	R5
	2/15	. . . . ~ . . . . Y . . . E . . . N V	R5
	1/15	. . . . ~ . . . . E . . . . V	R5
	1/15	. . . . Y Y I . K Q N R . . . . V T . . . . T . . L	X4
	1/15	. . . . . Y I . T Q R R . . . . V T A . . . T	X4
	**PBMC at baseline**		
	Consensus	**C T R P Y Y I T K Q N R I H I G P G R A F V T T G Q I T G K I R E A H C**	
	3/15	. . . .	X4
	2/15	. . . . L	X4
	2/15	. . . . A	X4
	1/15	. . . . I . . . . T . . . . A . . L	X4
	1/15	. . . . I . . . . T . . . . A . R	X4
	3/15	. . . . N . . . T . R . . . . A	X4
	3/15	. . . . N . . . T . R . . . . A . . . . K	X4

Patient no.	No. of clones / total tested	Amino acid sequence of the V3 *env* region	Predicted genotype
**16 (continued)**			
**Plasma on cART**			
	Consensus	C T R P Y Y I I K R N R I H I G P G R T F V T T G Q I A G K L K E A H C	
	11/16	. . . .	X4
	1/16	. . . . S	X4
	1/16	. . . . E	X4
	1/16	. . . . K . . D	X4
	2/16	. . . . D	X4
**CD4$^+$ T-cells on cART**			
	Consensus	C T R P Y Y I I K Q N R I H I G P G R T F V T T G Q I A G K L K E A H C	
	3/10	. . . . R	X4
	2/10	. . . .	X4
	1/10	. . G	X4
	1/10	. . . . I R	X4
	2/10	. . . . N N N T R ~ K S . . . . A . F A . . D . I . . I R	R5
	1/10	. . . . N N N T R ~ K S . . . . A . F A . . . . I . G I R Q . .	R5
**CD14^{high} CD16$^-$ monocytes on cART**			
	Consensus	C S R P Y Y I L K Q N R I H I G P G R T F V T T G Q I A G K L K E A H C	
	12/15	. . . .	X4
	1/15	. . . . S	X4
	1/15	. . . . C	X4
	1/15	. . . . A	X4
**18**			
**CD4$^+$ T-cells on cART**			
	Consensus	C T R P N N N T R K S I H I ~ ~ G P G R T L Y A T G E I I G D I R Q A H C	
	5/14	. . . . ~ ~	R5
	2/14	. . . . ~ ~ . . . . N . . . . Y .	R5
	3/14	. . . . ~ ~ . . . . D	R5
	1/14	. . . . R . ~ ~	R5
	1/14	. . . . G . . . . . ~ ~	R5
	1/14	. . . . ~ ~ . . . K	R5
	1/14	. . . . ~ ~ . . . K . . . . N . . . . Y .	R5
**CD14^{high} CD16$^-$ monocytes on cART**			
	Consensus	C T R P N N N T R K D I H I ~ ~ R P G R A F Y A T ~ D I I R N I R Q A H C	
	11/21	. . . . ~ ~ . . . . ~	R5
	1/21	. . . . ~ ~ . . . . ~ . . . . R .	R5
	2/21	. . . . ~ ~ . . . . ~ . . . . . M	R5
	1/21	. . . . ~ ~ . . . . . . H . . ~	R5
	1/21	. . . . S . R . Q R G . . . . . V T I G K ~ . G . M	X4
	3/21	. . . . . I . . . . S . R . Q R G . . . . . V T I G K ~ . G . M	X4
	1/21	. . . . S I . . . . S . R . Q R G . . . . . V T I G K ~ . G . M	X4
	1/21	. . . . S . C . Q R G . . . . . V T I G K ~ . G . M	X4
**19**			
**PBMC at baseline**			
	Consensus	C T R P N N N T R R S V P I ~ ~ G P G R A F Y T S ~ E I I G N I R Q A H C	R5
	8/13	. . . . ~ ~ . . . . ~	R5
	1/13	. . . . I . . ~ ~ . . . . ~	R5
	1/13	. . . . I . . ~ ~ . . E . . . . . . ~	R5
	2/13	. . . . H . ~ ~ . . . . ~	R5
	1/13	. . . . ~ ~ . . . . ~ . . . . R	R5
**CD4$^+$ T-cells on cART**			
	Consensus	C T R P N N N T R R S V P I ~ ~ G P G R A F Y T S ~ E I I G N I R Q A H C	
	7/11	. . . . ~ ~ . . . . ~	R5
	2/11	. . . . S . ~ ~ . . . . ~	R5
	2/11	. . . . ~ ~ . . . . ~ . T	R5
**CD14^{high} CD16$^-$ monocytes**			
	Consensus	C T R P N N N T R K R I R I Q R G P G R A F V T I G ~ K I G N M R Q A H C	
	17/20	. . . . ~	X4
	1/20	. . . . . . S . . . . ~	X4
	1/20	. . G . . . . ~	X4
	1/20	. . . . R . . . . ~	X4
**20**			
**PBMC at baseline**			
	Consensus	C T R P G N N T R K S V H I G P G R A F Y A T G D I I G D I R Q A H C	
	14/16	. . . .	R5
	1/16	. . . . C	R5
	1/16	. . . . E	R5
**Plasma on cART**			
	Consensus	C T R P G N N T R K S V H I G P G R A F Y A T G D I I G D I R R A H C	
	10/12	. . . .	R5
	1/12	. . . . V	R5
	1/12	. . . . . S	R5
**CD4$^+$ T-cells on cART**			
	Consensus	C T R P G N N T R K S V H I G P G R A F Y A T G D I I G D I R Q A H C	
	7/11	. . . .	R5
	2/11	. . . . T . . . . K . . .	R5
	2/11	. . . . R . . .	R5

Patient no.	No. of clones / total tested	Amino acid sequence of the V3 *env* region	Predicted genotype
**22**			
**PBMC at baseline**			
	Consensus	**C T R P N N N T R K G I H L G P G G A F Y A T G E I I G N I R Q A H C**	
	11/16	. . . .	**R5**
	1/16	. . . . **A**	**R5**
	1/16	. . . . **R**	**R5**
	1/16	. . . . **R**	**R5**
	1/16	. . . . **R** . . .	**R5**
	1/16	. . . . **R**	**R5**
**CD4$^+$ T-cells on cART**			
	Consensus	**C M R P G N N T R K S I S I G P G R A F Y A T G D I I G N I R Q A H C**	
	6/13	. . . .	**R5**
	2/13	. . . . **T**	**R5**
	3/13	. **I** . . . . **T**	**R5**
	1/13	. **I** . . . . **D**	**R5**
	1/13	. **T** . . . . **M R** . . . . **Q** . . . . **D T**	**R5**
**CD16$^+$ monocytes on cART**			
	Consensus	**C T R P N N N T R K G I H L G P G G A F Y T T G E I I G D I R Q A H C**	
	20/24	. . . .	**R5**
	4/24	. . . . **A** . . . . . . **N**	**R5**
**25**			
**Plasma on cART**			
	Consensus	**C T R P N N N T R K S I H F G P G R A L Y A T G N I I G D I R Q A H C**	
	9/12	. . . .	**R5**
	1/12	. **A**	**R5**
	1/12	. . . . **G**	**R5**
	1/12	. . . . **R**	**R5**
**CD4$^+$ T-cells on cART**			
	Consensus	**C T R P N N N T R K S I H F G P G R A L Y A T G N I I G D I R Q A H C**	
	8/10	. . . .	**R5**
	1/10	. . . . **E**	**R5**
	1/10	. . . . **N**	**R5**
**29**			
**Plasma at baseline**			
	Consensus	**C T R P N N N T R K G I H I G P G R A F Y A T E S I V G N I R Q A H C**	
	11/13	. . . .	**R5**
	1/13	. . . . **T**	**R5**
	1/13	. . . . **T**	**R5**
**PBMC at baseline**			
	Consensus	**C T R P N N N T R K G I H I G P G R A F Y A T E S I V G N I R Q A H C**	
	8/11	. . . .	**R5**
	1/11	. . . . **N**	**R5**
	1/11	. . . . **M**	**R5**
	1/11	. . . . **L**	**R5**
**Plasma on cART**			
	Consensus	**C T R P N N N T R K G I R I G P G R A F Y A T E R I V G N I R Q A H C**	
	8/13	. . . .	**X4**
	1/13	. . . . . **S**	**X4**
	1/13	. . . . **S**	**X4**
	1/13	**R**	**X4**
	1/13	. . . . **K**	**X4**
	1/13	. . . . **T** . . . . **R**	**X4**
**CD4$^+$ T-cells on cART**			
	Consensus	**C T R P N N N T R K G I H I G P G R A F Y A T E R I V G N I R Q A H C**	
	6/12	. . . .	**X4**
	6/12	. . . . **S**	**R5**
**CD14^{high} CD16$^-$ monocytes on cART**			
	Consensus	**C T R P S N N T R T G I H M G P G R T L Y A T G A I T G N I R K A Y C**	
	12/13	. . . .	**R5**
	1/13	. . . . **A**	**R5**
**CD16$^+$ monocytes on cART**			
	Consensus	**C T R P N N N T R K G I H I G P G R A F Y A T E S I V G N I R Q A H C**	
	11/12	. . . .	**R5**
	1/12	. . . . **M**	**R5**
